# Antibody-drug conjugates targeting the cadherin, claudin and nectin families of adhesion molecules

**DOI:** 10.3389/fmmed.2025.1661016

**Published:** 2025-10-14

**Authors:** Masuko Katoh, Yohann Loriot, Izuma Nakayama, Akinobu Hamada, Kohei Shitara, Masaru Katoh

**Affiliations:** ^1^ Department of Global Network, M & M Precision Medicine, Tokyo, Japan; ^2^ Drug Development Department (DITEP), Villejuif, France; ^3^ INSERM U981, Institut Gustave Roussy, Université Paris-Saclay, Villejuif, France; ^4^ Department of Gastroenterology and Gastrointestinal Oncology, National Cancer Center Hospital East, Kashiwa, Japan; ^5^ Division of Molecular Pharmacology, National Cancer Center, Tokyo, Japan; ^6^ Department of Pharmacology and Therapeutics, National Cancer Center, Tokyo, Japan; ^7^ Department of Omics Network, National Cancer Center, Tokyo, Japan

**Keywords:** alternative splicing, fibroblast growth factor receptor, gastric cancer, gene amplification, immune checkpoint inhibitor, loss of antigen or epitope, transdifferentiation, tumor heterogeneity

## Abstract

The classical cadherin (CDH), claudin (CLDN) and nectin families of transmembrane-type adhesion molecules are located at adherens or tight junctions in epithelial cells but diffuse to the nonjunctional cell surface in solid tumors with epithelial–mesenchymal plasticity. Human/humanized antibody-drug conjugates (ADCs) with chemical linkers and cytotoxic payloads have been developed for the treatment of malignancies. Here, the clinical development of ADCs that target CDH6, CDH17, CLDN6, CLDN18.2 and NECTIN4 is reviewed. Enfortumab vedotin is an NECTIN4-targeting antibody-drug conjugate that is approved for the treatment of urothelial cancer, whereas other ADCs or derivatives that target NECTIN4, such as bulumtatug fuvedotin, SHR-A2102 and zelenectide pevedotin, are being studied in randomized phase III clinical trials. In contrast, arcotatug tavatecan, garetatug rezetecan, sonesitatug vedotin and tecotabart vedotin are anti-CLDN18.2 ADCs in phase III clinical trials for the treatment of CLDN18.2-positive gastric or gastroesophageal junction adenocarcinomas, and raludotatug deruxtecan is an anti-CDH6 ADC in a phase II/III clinical trial for the treatment of platinum-resistant ovarian cancer. ADCs that target cell-cell adhesion molecules are a rapidly emerging class of cancer therapeutics, and bispecific ADCs and longitudinal companion diagnostics are emerging to further improve the clinical benefits of conventional ADCs.

## 1 Introduction

**FIGURE 1:**
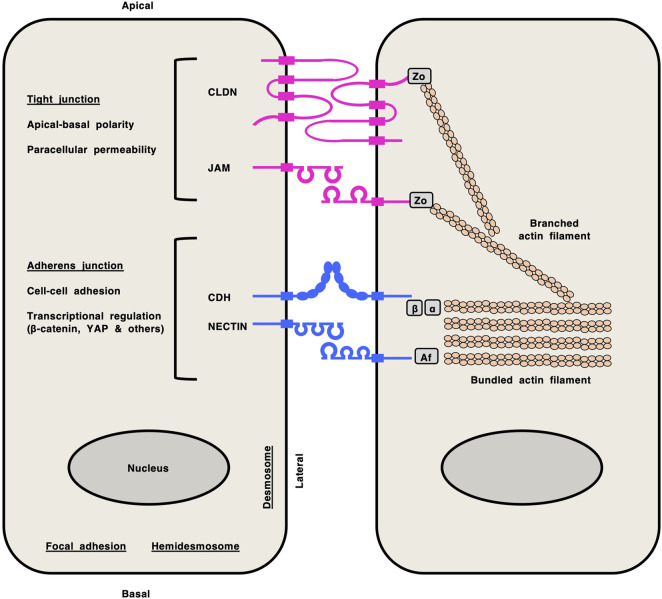
Cell-cell adhesion molecules in epithelial cells. Claudin (CLDN) and junctional adhesion molecule (JAM) at tight junctions are shown in pink, whereas classical cadherin (CDH) and NECTIN at adherens junctions are shown in blue. CLDNs and JAMs are tethered to cytoplasmic branched actin filaments via zonula occludens (Zo) proteins. Classical CDHs and NECTINs are tethered to cytoplasmic bundled actin filaments via the β-catenin (β)/α-catenin (α) complex and afadin (Af), respectively. Network of CLDN/JAM and CDH/NECTIN complexes via direct interactions of their extracellular regions and indirect interactions mediated by cytoplasmic scaffolding proteins regulates versatile functions in epithelial tissues, such as apical-basal polarity, paracellular permeability, cell-cell adhesion and transcription.

Classical cadherins (CDHs), claudins (CLDNs), junctional adhesion molecules (JAMs) and NECTINs are representative cell-cell adhesion molecules (Figure 1): classical CDHs and NECTINs are single-span transmembrane proteins that are present in adherens junctions and are tethered to cytoplasmic bundled actin filaments via the β-catenin/α-catenin complex and afadin, respectively (Gumbiner, 2005; Samanta and Almo, 2015; Takeichi, 2014); CLDNs and JAMs are tetra- and single-span transmembrane proteins, respectively, that are present in tight junctions and are tethered to cytoplasmic actin filaments via zonula occludens (ZO) proteins (Ebnet, 2017; Katoh and Katoh, 2024; Zihni et al., 2016). CDH/NECTIN and CLDN/JAM complexes network together via direct interactions of their extracellular regions and indirect interactions mediated by cytoplasmic scaffolding proteins, which dynamically regulate cell-cell adhesion, apical-basal polarity, transcription and paracellular permeability in epithelial tissues (Campbell et al., 2017; Horowitz et al., 2023; Karaman and Halder, 2018).

**FIGURE 2:**
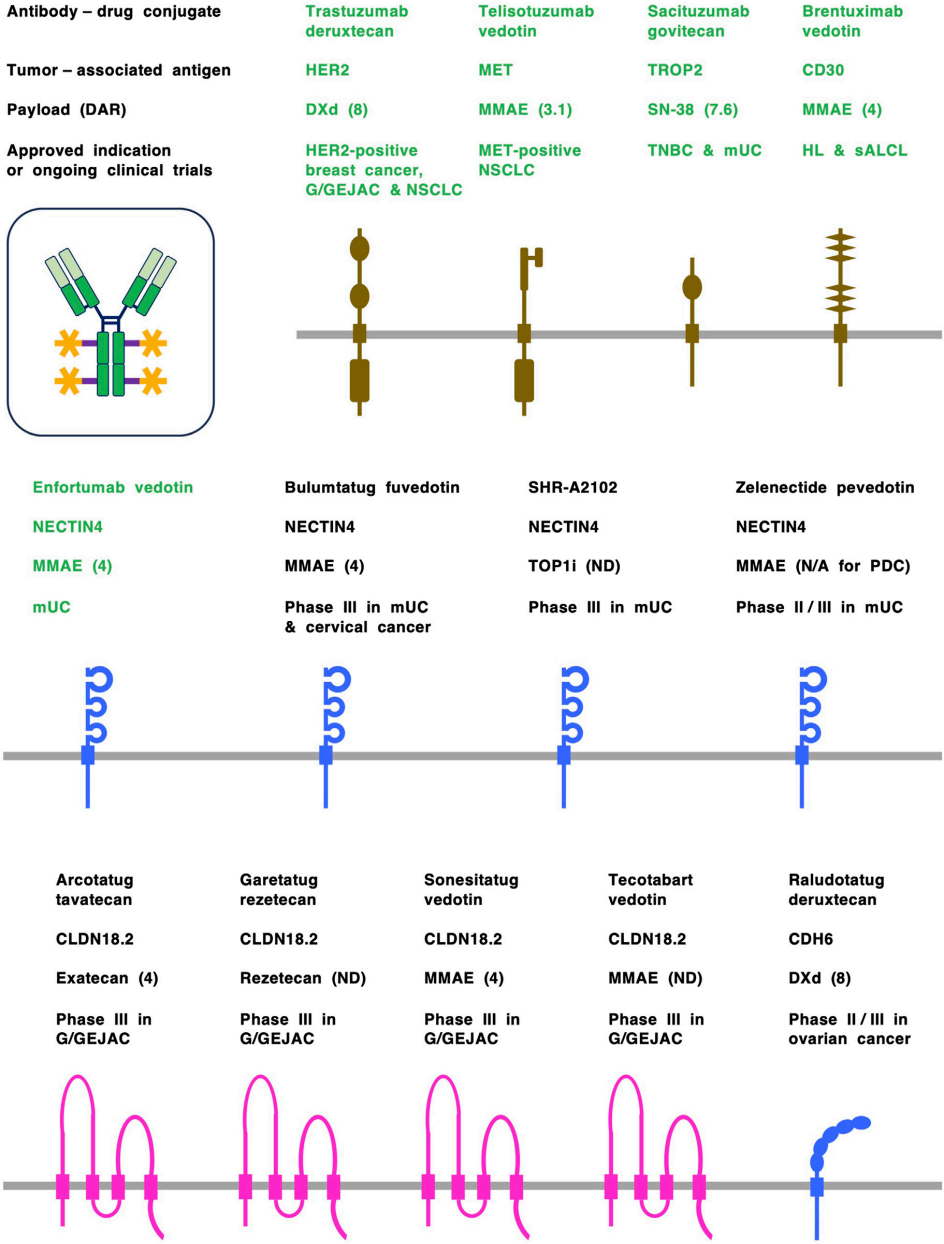
Antibody-drug conjugates (ADCs) and tumor-associated antigens (TAAs). ADCs are designed to preferentially deliver cytotoxic payloads to tumor cells through binding to TAAs and subsequent internalization. ADCs approved by the US Food and Drug Administration (FDA) are shown in green, whereas investigational ADCs in clinical trials are shown in black. TAAs derived from the cadherin (CDH) and NECTIN families of adrerens junction proteins are shown in blue, TAAs derived from the claudin (CLDN) family of tight junction proteins are shown in pink, and TAAs derived from receptor tyrosine kinases, etc., are shown in dark brown. Claudin 18 isoform 2 (CLDN18.2), human epidermal growth factor receptor 2 (HER2), MET, nectin cell adhesion molecule 4 (NECTIN4) and trophoblast cell surface antigen 2 (TROP2) are representative transmembrane-type TAAs on solid tumors, and CD30 is a transmembrane-type TAA on hematological malignancies. Each ADC consists of a human/humanized monoclonal antibody (mAb), a cleavable or non-cleavable chemical linker (purple) and a cytotoxic payload (orange). Monomethyl auristatin E (MMAE) is a microtubule disruptor, whereas deruxtecan (DXd), exatecan, rezetecan and SN-38 are topoisomerase I inhibitors (TOP1i). Drug-to-antibody rate (DAR) is shown in parentheses following payload. G/GEJAC, gastric or gastroesophageal junction adenocarcinoma; HL, Hodgkin’s lymphoma; mUC, metastatic urothelial cancer; N/A, not available; ND, not disclosed; NSCLC, non-small cell lung cancer; PDC, peptide-drug conjugate; sALCL, systemic anaplastic large cell lymphoma; TNBC, triple-negative breast cancer.

In contrast, antibody-drug conjugates (ADCs), each of which consists of a human/humanized monoclonal antibody (mAb), a chemical linker and a cytotoxic payload, are cutting-edge therapeutics that have been designed to increase antitumor activity and mitigate off-tumor adverse effects (Drago et al., 2021; Fu et al., 2022; Katoh and Katoh, 2020). Conventional ADCs targeting transmembrane proteins, such as human epidermal growth factor receptor 2 (HER2), MET, nectin cell adhesion molecule 4 (NECTIN4) and trophoblast cell surface antigen 2 (TROP2) (Figure 2), have been approved for the treatment of specific cancer types by the US Food and Drug Administration (FDA) and regulatory agencies in other countries (Colombo et al., 2024; Dumontet et al., 2023; FDA, 2025; Maecker et al., 2023).

The epithelial–mesenchymal plasticity of solid tumors is characterized by dynamic transitions among epithelial, intermediate (quasi-epithelial and quasi-mesenchymal) and mesenchymal cell states, which confer a variety of malignant features, such as invasion and metastasis, drug resistance and immune evasion (Dongre et al., 2022; Katoh and Katoh, 2022; Yang et al., 2020). Because NECTIN4, CLDNs and classical CDHs are located at cell-cell junctions in polarized epithelial tumor cells but diffuse to nonjunctional locations in contact-naïve intermediate or mesenchymal tumor cells (Ebnet, 2017; Nakayama et al., 2024), human/humanized ADCs with cleavable linkers and cutting-edge payloads that target these adhesion molecules are expected to be active on aggressive and/or heterogenous solid tumors with a spectrum of epithelial–mesenchymal transitions in part through bystander killing effects. Here, investigational and/or FDA-approved ADCs targeting NECTIN4, CLDN18.2, CLDN6, CDH6 and CDH17 will be overviewed, and then perspectives in this field will be discussed.

## 2 NECTIN4-targeting ADCs and ADC derivatives

NECTIN4 is an adherens junction protein with immunoglobulin-like extracellular domains and an afadin-binding cytoplasmic tail (Reymond et al., 2001). Physiologically, it is involved in cell-cell adhesion, but this protein has also been implicated as the measles virus receptor on epithelial cells (Mühlebach et al., 2011) and as a ligand of the immune coinhibitory receptor TIGIT (T-cell immunoglobulin and ITIM domain) (Reches et al., 2020), pointing to an extended role in modulating tumor-immune interactions.

Recently, NECTIN4 was characterized as a tumor-associated antigen (TAA). Immunohistochemical surveys have demonstrated moderate-to-strong NECTIN4 expression in 60.3% of bladder cancer cases (316/524), 52.3% of breast cancer cases (342/654), 36.6% of pancreatic cancer cases (60/164), 27.0% of lung cancer cases (167/618), 24.3% of esophageal cancer cases (44/181), 18.5% of head and neck cancer (HNC) cases (25/135) and 17.8% of ovarian cancer cases (21/118) (Challita-Eid et al., 2016). Given this increased expression, anti-NECTIN4 ADCs were trialed in bladder, breast, lung and pancreatic cancers and have demonstrated responses in preclinical xenograft models (Challita-Eid et al., 2016). These preclinical results have led to clinical trials of NECTIN4-targeting ADCs or ADC derivatives as listed in Table 1.

**TABLE 1:** NECTIN4-targeting ADCs or ADC derivatives.

Modality	Drug	Phase	Clinical trial ID	Design	Patients and results	Status
ADC	Enfortumab vedotin*	I	NCT02091999	Mono	Uro, ORR 43%	Completed
		I/II	NCT03288545	Combo ICI	Uro, ORR 73%	Active NR
		II	NCT03219333	Mono	Uro, ORR 44%	Completed
		II	NCT04225117	Mono	HNC, ORR 24%; Breast, ORR 17%; Eso and Gas, ORR 14%; NSCLC, ORR 11%	Active, NR
		III	NCT03474107	Mono vs. C	Uro, mOS 12.88 vs. 8.97, mPFS 5.55 vs. 3.71	Active NR
		III	NCT04223856	Combo ICI vs. C	Uro, mOS 31.5 vs. 16.1, mPFS 12.5 vs. 6.3	Active NR
ADC	Bulumtatug fuvedotin (9MW2821)	I/II	NCT05216965	Mono	Uro, ORR 54%; TNBC, ORR 50%; Cer, ORR 32%; Eso, ORR 14%	Recruiting
		III	NCT06196736	Mono vs. C	Uro	Recruiting
		III	NCT06692166	Mono vs. C	Cer	Recruiting
ADC	SHR-A2102	I	NCT05701709	Mono	Breast, ORR 60%; HNC, ORR 50%; NSCLC, ORR 33%	Active NR
		I	NCT05735275	Mono	Uro, ORR 38%	Recruiting
		II	NCT06654440	Mono	Gyne	Recruiting
		III	NCT06738251	Mono vs. C	Uro	Recruiting
ADC	ADRX-0706	I	NCT06036121	Mono	Solid tumors	Recruiting
ADC	IPH4502	I	NCT06781983	Mono	Solid tumors	Recruiting
ADC	LY4052031	I	NCT06465069	Mono	Solid tumors	Recruiting
ADC	LY4101174 (ETx-22)	I	NCT06238479	Mono	Solid tumors	Recruiting
PDC	Zelenectide pevedotin (BT8009)	I/II	NCT04561362	Mono or combo ICI	Uro Mono, ORR 50%; TNBC Mono, ORR 13%	Recruiting
		II/III	NCT06225596	Mono or combo ICI vs. C or C/ICI	Uro	Recruiting

* FDA approval for the treatment of urothelial cancer patients; Active NR, active, not recruiting; ADC, antibody-drug conjugate; Breast, hormone receptor-positive, HER2-negative breast cancer and triple-negative breast cancer; C, chemotherapy; Cer, cervical cancer; Combo, combination therapy; Eso, esophageal cancer; Gas, gastric cancer; Gyne, gynecological cancers; HNC, head and neck cancer; ICI, immune checkpoint inhibitor; Mono, monotherapy; mOS, median overall survival in months; mPFS, median progression free survival in months; NSCLC, non-small cell lung cancer; ORR, objective response rate; PDC; peptide-drug conjugate; TNBC, triple-negative breast cancer; Uro, urothelial cancer. Details of design and key endpoint(s) of each clinical trial with indicated ID are available at https://clinicaltrials.gov.

### 2.1 Enfortumab vedotin

Enfortumab vedotin is the first-generation NECTIN4-targeting ADC that is composed of a fully human anti-NECTIN4 mAb, a valine-citrulline cleavable linker and a microtubule-disrupting monomethyl auristatin E (MMAE) payload with a drug-to-antibody ratio (DAR) of 4 (Challita-Eid et al., 2016).

A phase I clinical trial of enfortumab vedotin in patients with NECTIN4-expressing solid tumors who progressed on chemotherapy and/or immune checkpoint inhibitor (ICI) therapy (EV-101 study, NCT02091999) revealed manageable tolerability and clinical activities with an objective response rate (ORR) of 42.9% (48/112) and a median overall survival (mOS) of 12.3 months in metastatic urothelial cancer patients (Rosenberg et al., 2020). In this EV-101 study, NECTIN4 expression was initially required for enrollment, but was then removed owing to NECTIN4 overexpression in almost all biopsied samples derived from metastatic urothelial cancers.

A phase II clinical trial of enfortumab vedotin in locally advanced or metastatic urothelial cancer patients with previous platinum-based chemotherapy and ICI therapy (EV-201 study, NCT03219333) also showed manageable tolerability with an ORR of 44.0% (55/125) and a complete response (CR) rate of 12.0% (Rosenberg et al., 2019). FDA accelerated approval was granted for the use of enfortumab vedotin in the treatment of metastatic urothelial cancer patients on the basis of this single-arm registry trial.

In the confirmatory phase III randomized clinical trial in urothelial cancer patients with previous platinum-based and ICI therapies (EV-301 study, NCT03474107), enfortumab vedotin achieved statistically significant and clinically meaningful improvement compared with investigator-chosen chemotherapy: mOS, 12.88 months *versus* 8.97 months [hazard ratio (HR), 0.70; 95% confidence interval (CI), 0.56 to 0.89; P = 0.001]; and median progression-free survival (mPFS), 5.55 months *versus* 3.71 months [HR, 0.62; 95% CI, 0.51 to 0.75; P < 0.001] (Powles et al., 2021). FDA regular approval was then granted to enfortumab vedotin for urothelial cancer patients in advanced-line settings.

While these clinical activities of enfortumab vedotin are encouraging, the proportion of complete responses were modest, and durable remissions remain limited with many patients still progressing within 6–12 months. This would suggest that NECTIN4 targeting might improve but not transform the natural history of advanced disease.

On the basis of these landmark results of enfortumab vedotin monotherapy for urothelial cancer, phase II clinical trials for the treatment of patients with breast cancer, esophageal cancer, gastric cancer, HNC and non-small cell lung cancer (NSCLC) (EV-202 study, NCT04225117), adenoid cystic carcinoma (NCT06891560), colorectal cancer or hepatocellular carcinoma (NCT06553885), pancreatic cancer (NCT05915351) and prostate cancer (NCT04754191) are ongoing to expand the indication of enfortumab vedotin monotherapy to other types of NECTIN4-positive cancers (Table 1). In the EV-202 study, cohorts of breast cancer (Giordano et al., 2024), gastroesophageal cancer (Muro et al., 2024a), HNC (Swiecicki et al., 2025) and NSCLC (Muro et al., 2024b) revealed manageable safety profiles and ORRs of 17% (15/87), 14% (12/86), 24% (11/46) and 11% (7/66), respectively. These results of weaker efficacy (ORRs 10%–20%) in non-urothelial tumors suggest that patient selection still remains an unresolved issue as despite ubiquitous NECTIN4 expression in urothelial tumors, this biomarker-based enrichment added little predictive value. This attenuated benefit highlights the need for better predictive biomarkers beyond expression alone (e.g., differential dependency, internalization kinetics, or tumor microenvironment factors).

The most compelling development has been a new frontline standard of enfortumab vedotin plus pembrolizumab due to synergy with checkpoint inhibitors. The phase Ib/II EV-103 study (NCT03288545) showed that combining enfortumab vedotin with pembrolizumab almost doubled ORRs of 64%–73% in front-line urothelial cancer to what would be expected from either monotherapy (Hoimes et al., 2023; O'Donnell et al., 2023). Following the accelerated approval of combination therapy by the FDA, the phase III EV-302 study (NCT04223856) confirmed the major survival advantage (mOS 31.5 months vs. 16.1 months with chemotherapy) with manageable toxicity (Powles et al., 2024). Mechanistically, the synergy may reflect immunogenic cell death triggered by MMAE payload release, enhancing anti-tumor immunity.

The strength of NECTIN4 as a target is in its cell-surface abundance and accessibility, making it highly suitable for ADC delivery in selected tumors. The benefit in urothelial cancer demonstrates the therapeutic potential but the variable efficacy in non-urothelial cancers suggest need of more validated predictive biomarkers beyond expression and improvements in combinations (e.g., immune checkpoint inhibitors), next-generation payloads, or bispecific ADCs.

### 2.2 Other NECTIN4-targeting ADCs and derivatives

Investigational ADCs or ADC derivatives targeting NECTIN4, such as bulumtatug fuvedotin (9MW2821) (Zhang J et al., 2025; Zhou et al., 2023), ADRX-0706 (Hau et al., 2024), IPH4502 (Sen et al., 2025), LY4052031 (Gao et al., 2025), LY4101174 (ETx-22) (Rosenberg et al., 2024), SHR-A2102 (Tang et al., 2025) and zelenectide pevedotin (BT8009) (Baldini et al., 2023; Loriot et al., 2025), have also entered clinical trials (Table 1).

Bulumtatug fuvedotin is an anti-NECTIN4 ADC conjugated with MMAE (the payload) via a valine-citrulline cleavable linker that has a stabilized and homogeneous DAR of 4 (Zhou et al., 2023). A phase I/II clinical trial of bulumtatug fuvedotin for solid tumor patients (NCT05216965) revealed ORRs of 54% in urothelial cancer, 50% in triple-negative breast cancer, 32% in cervical cancer and 14% in esophageal cancer (Zhang J et al., 2025), and randomized phase III clinical trials of bulumtatug fuvedotin *versus* chemotherapy in urothelial cancer patients (NCT06196736) and cervical cancer patients (NCT06692166) are ongoing.

SHR-A2102 is a NECTIN4-targeting ADC with a cleavable linker and a topoisomerase I inhibitor (TOP1i) payload that led to an ORR of 38% (28/73) in urothelial cancer patients in a phase I clinical trial in solid tumor patients (NCT05735275) (Tang et al., 2025), and ORRs of 60% (31/52), 50% (6/12) and 33.1% (52/157) in breast cancer, HNC and NSCLC patients, respectively, in another phase I clinical trial (NCT05701709) (Zhong R. et al., 2025). A phase II clinical trial of SHR-A2102 in gynecological cancer patients (NCT06654440) and a randomized phase III clinical trial of SHR-A2102 *versus* investigator-selected chemotherapy in urothelial cancer patients (NCT06738251) are ongoing.

ADRX-0706, IPH4502, LY4052031 and LY4101174 are next-generation anti-NECTIN4 ADCs with DARs of 8 (Hau et al., 2024; Sen et al., 2025; Gao et al., 2025; Rosenberg et al., 2024) that are currently in phase I clinical trials for the treatment of solid tumors (NCT06036121, NCT06781983, NCT06465069 and NCT06238479, respectively).

Zelenectide pevedotin is an ADC derivative of the peptide-drug conjugate that consists of a NECTIN4-binding bicyclic peptide, a valine-citrulline cleavable linker and an MMAE payload with a peptide-to-drug ratio of 1 (Rigby et al., 2022). Because of its lower molecular weight of approximately 4 kDa, high systemic *C*max value and ability to perform protease-dependent cleavage in the tumor microenvironment (TME), zelenectide pevedotin was expected to improve tumor penetration, enhance clinical efficacy and mitigate adverse events. In an ongoing phase I/II clinical trial for solid tumor patients (NCT04561362), zelenectide pevedotin a revealed manageable safety profile and preliminary ORRs of 50% (4/8) in urothelial cancer patients (Baldini et al., 2023) and 13% (4/30) in triple-negative breast cancer patients (Klümper et al., 2025). A phase II/III clinical trial of zelenectide pevedotin monotherapy or combination therapy with pembrolizumab *versus* chemotherapy for the treatment of urothelial cancer is also ongoing (Loriot et al., 2025).

## 3 CLDN-targeting ADCs

The 26 human CLDN proteins encoded by 24 human genes are tight junction proteins that consist of four transmembrane domains, cytoplasmic N- and C-terminal regions, a cytoplasmic loop and two extracellular loops (Katoh and Katoh, 2024). Investigational mAbs targeting the first or second extracellular loops of CLDNs have been generated for preclinical studies, and some of them have been tested in clinical trials (Chen et al., 2023; Nakayama et al., 2024; Vonniessen et al., 2024).

The clinical benefits of the mouse/human chimeric anti-CLDN18.2 mAb zolbetuximab (IMAB362) were demonstrated in the randomized phase III clinical trials GLOW (NCT03653507) and SPOTLIGHT (NCT03504397) (Shah et al., 2023; Shitara et al., 2023), and the FDA approved zolbetuximab plus chemotherapy for the treatment of gastric or gastroesophageal junction adenocarcinoma (G/GEJAC).

The clinical activities of zolbetuximab have led to the rapid emergence of human/humanized anti-CLDN mAbs and other CLDN-targeted therapies such as ADCs, bispecific antibodies and chimeric antigen receptor T (CAR-T) cell modalities (Katoh and Katoh, 2024; Nakayama et al., 2024). CLDN18.2 and CLDN6 are representative TAAs that can be clinically targeted with ADCs (Table 2).

**TABLE 2:** Claudin (CLDN)-targeting ADCs.

Target	Drug	Phase	Clinical trial ID	Design	Patients and results	Status
CLDN18.2	Arcotatug tavatecan (IBI343)	I	NCT05458219	Mono	G/GEJAC, ORR 32%; PDAC, ORR 23%	Recruiting
		III	NCT06238843	Mono vs. C	G/GEJAC	Recruiting
CLDN18.2	Garetatug rezetecan (SHR-A1904)	I	NCT04877717	Mono	G/GEJAC, ORR 28%	Active NR
		I/II	NCT05277168	Mono	G/GEJAC and PDAC	Recruiting
		III	NCT06649292	Mono vs. C	G/GEJAC	Recruiting
CLDN18.2	Sonesitatug vedotin (AZD0901 or CMG901)	I	NCT04805307	Mono	G/GEJAC, ORR 28%	Completed
		II	NCT06219941	Mono	G/GEJAC or BTC	Recruiting
				Combo C	PDAC	
		III	NCT06346392	Mono vs. C	G/GEJAC	Recruiting
CLDN18.2	Tecotabart vedotin (LM-302)	I/II	NCT05161390	Mono	G/GEJAC, ORR 31%	Active NR
		I/II	NCT05188664	Combo ICI	Solid tumors	Active NR
		II	NCT05934331	Combo ICI	GI cancers	Recruiting
		III	NCT06351020	Mono vs. C	G/GEJAC	Recruiting
CLDN18.2	Ciletatug vedotin (RC118)	I/II	NCT05205850	Mono	G/GEJAC ORR 47%	Recruiting
CLDN18.2	XNW27011	I/II	NCT06792435	Mono	G/GEJAC ORR 51%	Recruiting
CLDN18.2	ATG-022	I	NCT05718895	Mono	Gas, ORR 42%	Recruiting
CLDN18.2	EO-3021 (SYSA 1801)	I	NCT05009966	Mono	Gas/PDAC ORR 38%	Unknown
		I	NCT05980416	Mono and Combo	Solid tumors	Terminated
CLDN18.2	JS107	I	NCT05502393	Mono and Combo	G/GEJAC ORR 35%	Unknown
CLDN18.2	TORL-2-307-ADC	I	NCT05156866	Mono	Solid tumors	Recruiting
CLDN18.2	TQB2103	I	NCT05867563	Mono	Solid tumors	Unknown
CLDN6	Ixotatug vedotin (TORL-1-23)	I	NCT05103683	Mono	Solid tumors, ORR 33%	Recruiting
		II	NCT06690775	Mono	PROC	Recruiting
CLDN6	DS-9606a	I	NCT05394675	Mono	Solid tumors	Recruiting
CLDN6	QLS5132	I	NCT06932094	Mono	Solid tumors	Not yet

Active NR, active, not recruiting; ADC, antibody-drug conjugate; BTC, biliary tract cancer, C, chemotherapy; Combo, combination therapy; G/GEJAC, gastric or gastroesophageal junction adenocarcinoma; Gas, gastric cancer; GI, gastrointestinal; ICI, immune checkpoint inhibitor; Mono, monotherapy; Not yet, not yet recruiting; ORR, objective response rate; PDAC, pancreatic ductal adenocarcinoma; PROC, platinum-resistant ovarian cancer. Details of design and key endpoint(s) of each clinical trial with indicated ID are available at https://clinicaltrials.gov.

### 3.1 CLDN18.2-targeting ADCs

The *CLDN18* gene at human chromosome 3q22.3 encodes CLDN18.1 and CLDN18.2 isoforms that are expressed in the lung and stomach, respectively, on the basis of alternative promoters (Niimi et al., 2001). CLDN18.1 and CLDN18.2 are almost identical expect for the N-terminal cytoplasmic region, the first transmembrane domain and a part of the first extracellular loop that are derived from alternative exons 1a and 1b, respectively (Katoh et al., 2024b). CLDN18.2 is orthotopically overexpressed in 27–56% of gastric adenocarcinomas (Dottermusch et al., 2019; Nakayama et al., 2024; Sahin et al., 2008) and ectopically overexpressed in 50% of esophageal adenocarcinomas (Sahin et al., 2008), 30–60% of pancreatic ductal adenocarcinomas (PDACs) (Lyu et al., 2024; Sahin et al., 2008), 10% of ovarian adenocarcinomas (Sahin et al., 2008) and 4% of NSCLCs (Micke et al., 2014).

ATG-022 (Ma et al., 2025), ciletatug vedotin (RC118) (Liu T et al., 2024), EO-3021 (CPO102 or SYSA 1801) (Wang Y. et al., 2023), JS107 (Xu R. H. et al., 2025), sonesitatug vedotin (AZD0901 or CMG901) (Xu G. et al., 2024; Ruan et al., 2025), tecotabart vedotin (LM-302, BMS-986476 or TPX-4589) (Huang W et al., 2022; Bai C et al., 2024) and TORL-2-307-ADC (O'Brien et al., 2023) are human/humanized anti-CLDN18.2 ADCs with an MMAE payload, while arcotatug tavatecan (IBI343) (Liu J. J et al., 2024; Yu X. et al., 2025b; Shen et al., 2025), garetatug rezetecan (SHR-A1904) (Xu R. H. et al., 2024), TQB2103 (Cheng X et al., 2025) and XNW27011 (Yu J. et al., 2024 and 2025) are CLDN18.2-targeting ADCs with TOP1i payloads. All of these investigational anti-CLDN18.2 ADCs are being tested in clinical trials (Table 2).

Sonesitatug vedotin consists of a humanized anti-CLDN18.2 immunoglobulin G subtype 1 (IgG1) mAb, a cleavable linker and an MMAE payload with a DAR of 4 (Xu G. et al., 2024). Sonesitatug vedotin has revealed direct killing effects on CLDN18.2-overexpressing cancer cells via MMAE-dependent cytotoxicity, antibody-dependent cellular cytotoxicity (ADCC) and complement-dependent cytotoxicity (CDC) as well as indirect killing effects on bystander cancer cells through MMAE release from sonesitatug vedotin-internalized cells, which results in antitumor activities in preclinical models of gastric and pancreatic cancers (Xu G. et al., 2024). A phase I clinical trial of sonesitatug vedotin in patients with advanced solid tumors (KYM901 study, NCT04805307) revealed manageable safety profiles despite dose reduction and discontinuation owing to treatment-emergent adverse events in 14% and 7%, respectively, and promising clinical activities, such as a confirmed ORR of 28% (32/113) in G/GEJAC patients and a confirmed ORR of 33% (31/93) in CLDN18.2-high subgroup, which was defined as “CLDN18.2 membrane staining of 2+/3+ intensity in at least 20% of tumor cells using a diagnostic antibody EPR19202-244 instead of the standard diagnostic antibody 43-14A” (Ruan et al., 2025). A phase II clinical trial of sonesitatug vedotin monotherapy for CLDN18.2-positive G/GEJAC or biliary tract cancer patients and the combination of sonesitatug vedotin plus chemotherapy for CLDN18.2-positive PDAC patients (CLARITY-PanTumor01 study, NCT06219941) as well as a phase III randomized clinical trial of sonesitatug vedotin monotherapy *versus* the investigator’s choice of therapy for CLDN18.2-positive G/GEJAC patients in second- or later-line settings (CLARITY-Gastric 01 study, NCT06346392) are ongoing. The FDA’s fast-track designation was granted to sonesitatug vedotin monotherapy for the treatment of CLDN18.2-positive G/GEJAC (Rosa, 2023).

Tecotabart vedotin, which also consists of a humanized anti-CLDN18.2 IgG1 mAb, a cleavable linker and an MMAE payload, demonstrated superior antitumor efficacy to the mouse/human chimeric mAb zolbetuximab in a preclinical xenograft model of gastric cancer (Huang W et al., 2022). A phase I/II clinical trial of tecotabart vedotin for the treatment of advanced solid tumors (NCT05161390) revealed manageable safety and tolerability in a phase I part and a single-agent ORR of 31% (11/36) in CLDN18.2-positive G/GEJAC patients (Bai C et al., 2024). Phase I/II clinical trials of tecotabart vedotin plus immune checkpoint inhibitor toripalimab (NCT05188664 and NCT05934331) revealed ORR of 73% (24/33) in G/GEJAC or esophageal adenocarcinoma patients with CLDN18.2 staining of 2+/3+ intensity in at least 25% of tumor cells (Jiang et al., 2025). A phase III randomized clinical trial of tecotabart vedotin monotherapy *versus* the investigator’s choice of therapy for the treatment of CLDN18.2-positive G/GEJAC is ongoing (NCT06351020).

Among other CLDN18.2-targeting ADCs with an MMAE payload, phase I studies of ATG-022 (NCT05718895), EO-3021 (NCT05009966) and JS107 (NCT05502393) revealed ORRs of 42% (5/12) in gastric cancer patients (Ma et al., 2025), 38% (8/21) in gastric or pancreatic cancer patients (Wang Y. et al., 2023) and 35% (8/23) in CLDN18.2-high G/GEJAC patients (Xu R. H. et al., 2025), respectively, whereas a phase I/II clinical trial of ciletatug vedotin (NCT05205850) revealed an ORR of 47% (8/17) in CLDN18.2-positive G/GEJAC patients (Liu T et al., 2024).

In contrast, arcotatug tavatecan consists of an engineered anti-CLDN18.2 IgG1 mAb with fragment crystallizable (Fc) silencing, a cleavable linker and an exatecan payload with a DAR of 4, which was designed to reduce the risk of adverse events owing to defects in Fc-mediated ADCC (Liu J. J et al., 2024; Yu X. et al., 2025b). A phase I clinical trial of arcotatug tavatecan for the treatment of advanced solid tumors (NCT05458219) revealed manageable tolerability and clinical activity, including a single-agent ORR of 32% (32/99) in CLDN18.2-positive G/GEJAC patients (Liu J. J et al., 2024) and confirmed ORRs of 23% (10/44) *versus* 0% (0/12), mPFS of 5.4 *versus* 1.4 months and mOS of 9.1 *versus* 6.2 months in PDAC patients with CLDN18.2 staining in at least 60% *versus* less than 60% of tumor cells (Yu X. et al., 2025b). On the basis of these results, breakthrough therapy designation was granted to arcotatug tavatecan monotherapy for the treatment of CLDN18.2-positive G/GEJAC patients by the China National Medical Products Administration (NMPA), whereas fast-track designation was granted by the US FDA for the use of arcotatug tavatecan monotherapy in the treatment of PDAC (Wahner, 2024). A phase III randomized clinical trial of arcotatug tavatecan monotherapy *versus* the investigator’s choice of therapy (G-HOPE-001 study, NCT06238843) is recruiting G/GEJAC patients with CLDN18.2 membrane staining of 2+/3+ intensity in at least 75% of tumor cells (Shen et al., 2025).

Garetatug rezetecan, TQB2103 and XNW27011 are also anti-CLDN18.2 ADCs with TOP1i payloads. Garetatug rezetecan without disclosed DAR revealed a manageable safety profile and ORR of 28% (16/58) in CLDN18.2-positive G/GEJAC patients in a phase I clinical trial (NCT04877717) (Xu R. H. et al., 2024) and proceeded to phase I/II (NCT05277168) and phase III (NCT06649292) clinical trials. TQB2103 with a DAR of eight exhibited a favorable safety profile and ORRs of 20% (6/30) and 43% (3/7) in solid tumor patients with CLDN18.2 staining in at least 10% of tumor cells and CLDN18.2 staining of 2+/3+ intensity in at least 40% of tumor cells, respectively, in a phase I clinical trial (NCT05867563) (Cheng X et al., 2025). XNW27011, with a homogenous DAR of 8, showed a favorable safety profile in the dose escalation cohort of solid tumor patients (Yu J. et al., 2024) and ORRs of 31% (9/29), 61% (19/31) and 67% (12/18) in dose expansion cohorts of 2.4, 3.0 and 3.6 mg/kg doses, respectively, of G/GEJAC patients with CLDN18.2 staining of 2+/3+ intensity in at least 5% of tumor cells (Yu et al., 2025a) in a phase I/II clinical trial (NCT06792435). XNW27011 was licensed out to Astellas Pharma that manufactures and sells enfortumab vedotin and zolbetuximab (Plieth, 2025).

### 3.2 CLDN6-targeting ADCs

CLDN6 is expressed in pluripotent stem cells and fetal tissues but repressed in most adult tissues (Ben-David et al., 2013; Kong et al., 2021; Reinhard et al., 2020), whereas CLDN6 is overexpressed in 54–100% of germ cell tumors, 14–55% of ovarian cancers, 17–21% of endometrial cancers, 10–52% of gastric cancers and 6–11% of NSCLCs (Du et al., 2021; Micke et al., 2014; Qu et al., 2021; Zhang C. et al., 2021). The development of CLDN6-targeting biologics has become a trend on the basis of the potential of CLDN6 as a TAA (Katoh and Katoh, 2024).

Ixotatug vedotin (TORL-1-23) is a humanized anti-CLDN6 ADC with a conventional MMAE payload with a DAR of 4 (McDermott et al., 2023; Konecny et al., 2024), whereas AT65474 (Zhong C. et al., 2025), C6P (Yu X. et al., 2025a), DS-9606a (Patel et al., 2024), GB01-VA-PL2202 (Tsang et al., 2024), PLB-002 (Pan et al., 2025) and QLS5132 (Huang Y et al., 2025) are anti-CLDN6 ADCs with non-MMAE payloads. Among these investigational anti-CLDN6 ADCs, ixotatug vedotin, DS-9606a and QLS5132 are being tested in clinical trials (Table 2).

A phase I clinical trial of ixotatug vedotin for the treatment of advanced cancers (NCT05103683) revealed favorable safety profiles and preliminary efficacy across CLDN6-positive ovarian, endometrial and testicular cancer patients (Konecny et al., 2024). Notably, the ORR in patients treated with 0.2–3.0 mg/kg doses of ixotatug vedotin was 33% (21/64), and the ORRs in CLDN6-positive platinum-resistant ovarian cancer patients with 2.4 or 3.0 mg/kg ixotatug vedotin were 50% and 42%, respectively (Konecny et al., 2024). On the basis of promising clinical activities in the NCT05103683 clinical trial, a registry phase II clinical trial of ixotatug vedotin for the treatment of CLDN6-positive platinum-resistant epithelial ovarian cancer is ongoing (CATALINA-2 study, NCT06690775).

DS-9606a and QLS5132 are in phase I clinical trials (NCT05394675 and NCT06932094, respectively) for the treatment of advanced solid tumors (Table 2). DS-9606a revealed preliminary partial responses in four of 53 cancer patients (Patel et al., 2024), whereas QLS5132, which has a wide therapeutic window, is expected to exhibit superior efficacy and safety profiles in patients with CLDN6-positive ovarian cancers, gastric cancers, NSCLCs and other cancers (Huang Y et al., 2025).

## 4 CDH-targeting ADCs

CDH1 (E-cadherin, epithelial), CDH2 (N-cadherin, neuronal), CDH3 (P-cadherin, placental), CDH4 (R-cadherin, retinal), CDH5 (VE-cadherin, vascular endothelial) and CDH6 (K-cadherin, fetal kidney) are representative classical CDHs that contain five extracellular cadherin-repeat (EC) domains, a single transmembrane domain and a cytoplasmic region interacting with armadillo-repeat proteins, such as β-catenin and p120-catenin (Gumbiner, 2005; Takeichi, 2014), whereas CDH16 (KSP-cadherin, kidney specific) and CDH17 (LI-cadherin, liver-intestine) are nonclassical CDHs with seven EC domains, a single transmembrane domain and a truncated cytoplasmic region defective in catenin-binding motifs (Kreft et al., 1997; Wendeler et al., 2004). Because CDH6 (Köbel et al., 2008; Luo S. et al., 2021; Paul et al., 1997; Sato et al., 2025) and CDH17 (Cheng G et al., 2025; Jacobsen et al., 2024; O'Brien et al., 2024) have emerged as potential TAAs, CDH6- and CDH17-targeting ADCs have entered clinical trials (Table 3).

**TABLE 3:** Cadherin (CDH)-targeting ADCs.

Target	Drug	Phase	Clinical trial ID	Design	Patients and results	Status
CDH6	Raludotatug deruxtecan (DS-6000)	I	NCT04707248	Mono	PROC, ORR 38%	Active NR
		II	NCT06660654	Mono	Gyne, Uro and ccRCC	Recruiting
		II	NCT06864169	Mono	GI	Recruiting
		II/III	NCT06161025	Mono vs. C	Gyne	Active NR
CDH6	CUSP06	I	NCT06234423	Mono	PROC, ORR 36%	Recruiting
CDH6	HKT288	I	NCT02947152	Mono	ORR 0%	Terminated
CDH17	AMT-676	I	NCT06400485	Mono	Solid tumors	Recruiting
CDH17	TORL-3-600	I	NCT05948826	Mono	Solid tumors	Recruiting
CDH17	YL217	I	NCT06859762	Mono	Solid tumors	Not yet
CDH17	7MW4911	Preclin				
CDH17	BR116	Preclin				
CDH17	BSI-721	Preclin				
CDH17	LBL-054	Preclin				
CDH17	LM-350	Preclin				
CDH17	MRG007	Preclin				
CDH17	SCR-A008	Preclin				
CDH17	SOT109	Preclin				

Active NR, active, not recruiting; ADC, antibody-drug conjugate; C, chemotherapy; ccRCC, clear cell renal carcinoma; GI, gastrointestinal cancers; Gyne, gynecological cancers; Mono, monotherapy; Not yet, not yet recruiting; ORR, objective response rate; Preclin; preclinical stage; PROC, platinum-resistant ovarian cancer; Uro, urothelial cancer. Details of design and key endpoint(s) of each clinical trial with indicated ID are available at https://clinicaltrials.gov.

### 4.1 CDH6-targeting ADCs

CDH6 is a classical CDH that interacts with αIIbβ3 or α2β1 integrins and activates α2β1 integrin signaling to promote the invasion and metastasis of ovarian cancer and renal cell carcinoma (Bartolomé et al., 2021). Because CDH6 is upregulated in endometrial cancer, gastric cancer, ovarian cancer, pancreatic cancer, papillary thyroid cancer and renal cell carcinoma, CLDN6-targeting ADCs, such as CUSP06 (AMT-707), HKT288 and raludotatug deruxtecan (R-DXd or DS-6000a), have been developed for the treatment of cancer (Bialucha et al., 2017; Patel et al., 2025; Suzuki et al., 2024).

HKT288, a first-generation anti-CDH6 ADC with a microtubule-targeting DM4 payload, has shown durable preclinical activity in 40% of patient-derived xenograft (PDX) models, especially those derived from ovarian cancer and renal cell carcinoma (Bialucha et al., 2017). HKT288 entered a phase I clinical trial in patients with epithelial ovarian cancer and renal cell carcinoma (NCT02947152), because preclinical or coclinical studies of PDX models that harbor conserved characteristics of primary tumors could predict ADC activities in clinical trials (Yagishita et al., 2023). However, HKT288 exhibited unexpected neurological toxicity, and its clinical development was discontinued (Schöffski et al., 2021).

CUSP06 and raludotatug deruxtecan are second-generation CDH6-trgeting ADCs with TOP1i payloads, exatecan and exatecan derivative deruxtecan (DXd), respectively (Patel et al., 2025; Suzuki et al., 2024). Phase I clinical trials of CUSP06 (CUSP06-1001 study, NCT06234423) and raludotatug deruxtecan (NCT04707248) revealed ORRs of 36% (5/14) (Patel et al., 2025) and 38% (13/34) (Moore et al., 2023), respectively, in platinum-resistant ovarian cancer patients. The encouraging clinical activities and manageable safety profiles observed with these clinical trials led to the fast-track designation of CUSP06 by the FDA (Sava, 2025) and the launch of a phase II/III clinical trial of raludotatug deruxtecan (NCT06161025).

### 4.2 CDH17-targeting ADCs

CDH17 is a nonclassical CDH that interacts with the desmosomal cadherin desmocollin-1 (DSC1) and indirectly interacts with actin filaments via the DSC1/p120-catenin complex to promote the migration and invasion of colorectal cancer (Bartolomé et al., 2024). CDH17 is upregulated in 50–100% of colorectal cancer cases, 52–73% of gastric cancer cases, 61% of mucinous ovarian cancer cases, 53% of cervical adenocarcinoma cases, and 20–31% of pancreatic cancer cases (Cheng G et al., 2025; Jacobsen et al., 2024; O'Brien et al., 2024).

Recently, CDH17-targeting ADCs, including 7MW4911 (Wang R. et al., 2025), AMT-676 (Lemech et al., 2025), BR116 (Zhao et al., 2025), BSI-721 (Hao et al., 2025), LBL-054 (Ye et al., 2025), LM-350 (Huang W et al., 2025), MRG007 (Zeng et al., 2025), SCR-A008 (Cheng G et al., 2025), SOT109 (Kalynovska et al., 2025), TORL-3-600 (O'Brien et al., 2024) and YL217 (Lian et al., 2025), have been developed: BSI-721 and TORL-3-600 are humanized ADCs with an MMAE payload, whereas 7MW4911, AMT-676, BR116, LBL-054, LM-350, MRG007, SCR-A008, SOT109 and YL217 are human/humanized ADCs with TOP1i payloads; notably, the DARs of LM-350 and SCR-A008 are 8, and the DARs of 7MW4911, AMT-676, BR116 and BSI-721 are 4. Among these investigational anti-CDH17 ADCs, AMT-676, TORL-3-600 and YL217 have entered phase I clinical trials for the treatment of advanced cancers (NCT06400485, NCT05948826 and NCT06859762, respectively).

## 5 Perspectives

FDA-approved ADCs have drastically changed clinical practices for the treatment of urothelial cancer (Powles et al., 2021), HER2-positive breast cancer (Hurvitz et al., 2023) and CD30-positive peripheral T-cell lymphoma (Horwitz et al., 2022) and other cancer types (Figure 2). Nevertheless, several issues remain to be overcome to further enhance the anticancer effects and reduce the off-tumor effects of ADCs. Recent progress in antibody engineering, payload diversity, chemical linkers and conjugation technologies for the tumor-specific delivery of cytotoxic drugs with DARs of 4–8 (Colombo et al., 2024; Dumontet et al., 2023; Tsuchikama et al., 2024), adverse events of FDA-approved ADCs, such as neutropenia and interstitial lung disease or pneumonitis (Ballestín et al., 2025), and resistance mechanisms (Drago et al., 2021; Khoury et al., 2023; Li S et al., 2025) have been reviewed elsewhere, whereas progress regarding bispecific ADCs and longitudinal companion diagnostics are discussed here.

### 5.1 Bispecific ADCs targeting adhesion molecules

Bispecific ADCs are largely classified into ADCs that bind to dual epitopes on the same TAA (biparatopic) and ADCs that bind to dual epitopes on distinct TAAs (bispecific in a narrow sense) (Tsuchikama et al., 2024). Bispecific ADCs that can specifically bind to tumor cells and be efficiently internalized and processed for payload release have been developed with promise for improved clinical benefits and mitigated adverse effects.

**FIGURE 3:**
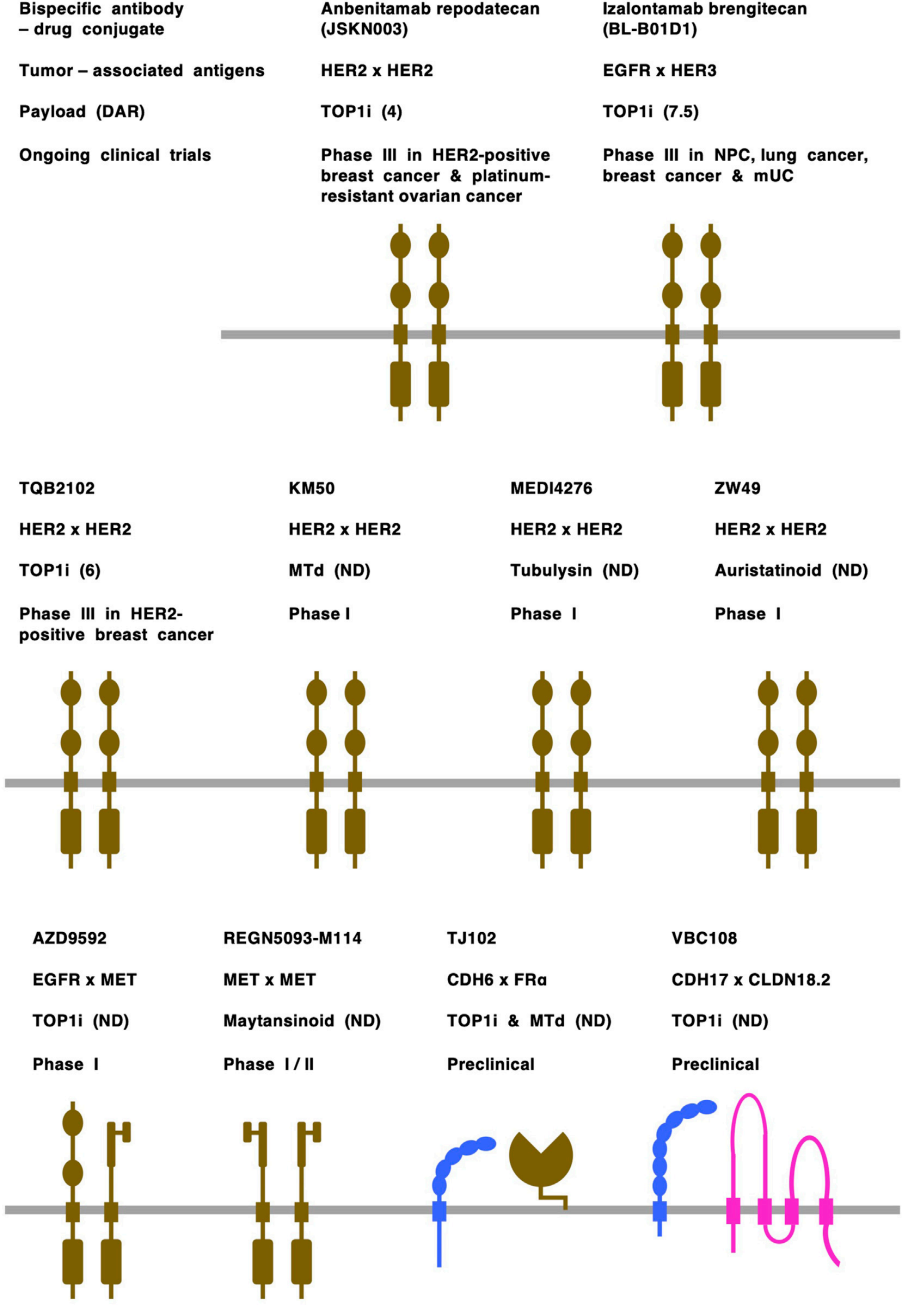
Bispecific antibody-drug conjugates (ADCs). Tumor-associated antigens: cadherin-6 (CHD6) and CDH17 are shown in blue; claudin 18 isoform2 (CLDN18.2) is shown in pink; and receptor tyrosine kinases, including epidermal growth factor receptor (EGFR), human EGFR2 (HER2), human EGFR3 (HER3) and MET, and folate receptor alpha (FRα) are shown in dark brown. Payloads: monomethyl auristatin E (MMAE), auristatinoid, maytansinoid and tubulysin are microtubule disruptors (MTd); other payloads are topoisomerase I inhibitors (TOP1i). Drug-to-antibody rate (DAR) is shown in parentheses following payload. mUC, metastatic urothelial cancer; ND, not disclosed; NPC, nasopharyngeal cancer.

Receptor tyrosine kinases (RTKs) constitute the major class of TAAs for bispecific ADCs (Figure 3), and those targeting HER2 x HER2 (anbenitamab repodatecan [JSKN003], TQB2102, KM501, MEDI4276 and ZW49), EGFR x HER3 (izalontamab brengitecan [BL-B01D1]), EGFR x MET (AZD9592) and MET x MET (REGN5093-M114) have entered clinical trials (Gu et al., 2024). Among RTK-targeting bispecific ADCs, anbenitamab repodatecan (Park et al., 2024) and TQB2102 (Li J et al., 2025) have been used in randomized phase III clinical trials for HER2-positive breast cancer patients (NCT06846437 and NCT06561607, respectively), and izalontamab brengitecan (Ma et al., 2024) has been assessed in phase III clinical trials for patients with nasopharyngeal carcinoma (NCT06118333), lung cancer (NCT06382116 and NCT06500026) and other types of cancers (NCT06304974, NCT06343948, NCT06382142 and NCT06857175).

Cell-cell adhesion molecules are emerging as a new class of TAAs for bispecific ADCs (Figure 3), and those targeting CDH3 x CDH17 (Synan et al., 2025), CDH6 x FRα (folate receptor alpha) (Zhang Y et al., 2025), CDH17 x CLDN18.2 (Wang W. V. et al., 2025) and CLDN3 x EpCAM (epithelial cell adhesion molecule) (Luo M. et al., 2025) have been shown to have anti-tumor effects on colorectal cancers, ovarian/kidney cancers, gastrointestinal cancers and solid tumors, respectively, in preclinical studies. In addition, on the basis of the overexpression of CLDN18.2 (Dottermusch et al., 2019; Nakayama et al., 2024; Sahin et al., 2008) and RTKs (Katoh et al., 2024a; Lee et al., 2012; Van Cutsem et al., 2015) in human gastric cancers, it was previously predicted that bispecific ADCs, such as CLDN18.2 x FGFR2 (fibroblast growth factor receptor 2), CLDN18.2 x HER2 and CLDN18.2 x MET, would be developed for the treatment of minor subsets of gastric cancer patients in the future (Katoh and Katoh, 2024).

Bispecific ADCs targeting adhesion molecules remain in the preclinical stage, probably owing to the lack of mechanistic understanding and clinical validation of TAA coexpression in primary tumor cells. Because TJ102, which targets both CDH6 and FRα (Zhang J et al., 2025), and VBC108, which bispecifically targets CDH17 and CLDN18.2 (Wang W. V. et al., 2025), demonstrated efficacy in mouse models and tolerability in cynomolgus monkeys, TJ102 and VBC108 are anticipated to enter clinical trials in patients with solid tumors, especially ovarian cancer and gastric cancer, respectively.

### 5.2 Longitudinal companion diagnostics

Immunohistochemical staining of NECTIN4 is not currently required for the use of the FDA-approved enfortumab vedotin (Klümper et al., 2023), owing to frequent NECTIN4 expression in urothelial tumors (immunohistochemical staining positive in 82.8% and strong/moderate staining in 60.3%) (Challita-Eid et al., 2016). However, because membranous immunohistochemical staining (Klümper et al., 2023), mRNA expression (Stewart et al., 2024) and gene amplification (Klümper et al., 2024) of NECTIN4 are predictive biomarkers of the enfortumab vedotin response, and because the *NECTIN4* mRNA level in urothelial cancer is upregulated in luminal subtypes but downregulated in the neuroendocrine-like subtype (Chu et al., 2021), companion diagnostics of enfortumab vedotin are emerging as a hot issue for the real-world management of urothelial cancer patients.

Immunohistochemical staining of CLDN18.2 is utilized as a selection biomarker for the treatment with investigational CLDN18.2-targeting ADCs because G/GEJAC patients with 2+/3+ staining in at least 20% *versus* less than 20% of tumor cells revealed ORRs of 33% *versus* 5%, respectively, in the KYM901 study of sonesitatug vedotin (Ruan et al., 2025). CLDN18.2 staining positivities on the basis of designated diagnostic antibody, immunohistochemical staining intensity and positive cell content are inclusion criteria for phase III clinical trials of anti-CLDN18.2 ADCs, such as arcotatug tavatecan (NCT06238843), sonesitatug vedotin (NCT06346392), garetatug rezetecan (NCT06649292) and tecotabart vedotin (NCT06351020), and may be applied as companion diagnostics in the future.

In contrast, positron emission tomography (PET) and single-photon emission computed tomography (SPECT) are medical imaging technologies that can detect primary tumors and metabolic lesions at the whole-body level (Voss, 2023; McGale et al., 2023). PET/CT hybrid imaging with radiolabeled NECTIN4-targeted bicyclic peptides, such as ^68^Ga-N188 and ^68^Ga-FZ-NR-1, was applied for urothelial cancer patients (Zhang J. et al., 2024; Sun et al., 2025), and PET/CT and SPECT/CT with radiolabeled CLDN18.2-targeted nanobodies, such as [^68^Ga]Ga-PMD22 and [^99^mTc]Tc-PHG102, respectively, were used for gastrointestinal cancer patients (Wang R. et al., 2024; Bai Z et al., 2024). In addition, CDH17-targeted hybrid imaging has been investigated in preclinical studies of PDAC and gastric cancer (Delaney et al., 2024; Mao et al., 2025). Investigational hybrid imaging features that are correlated with membranous immunohistochemical staining and can be used to detect primary, recurrent or metastatic tumor lesions are entering into clinical trials as noninvasive companion diagnostics of ADCs.

**FIGURE 4:**
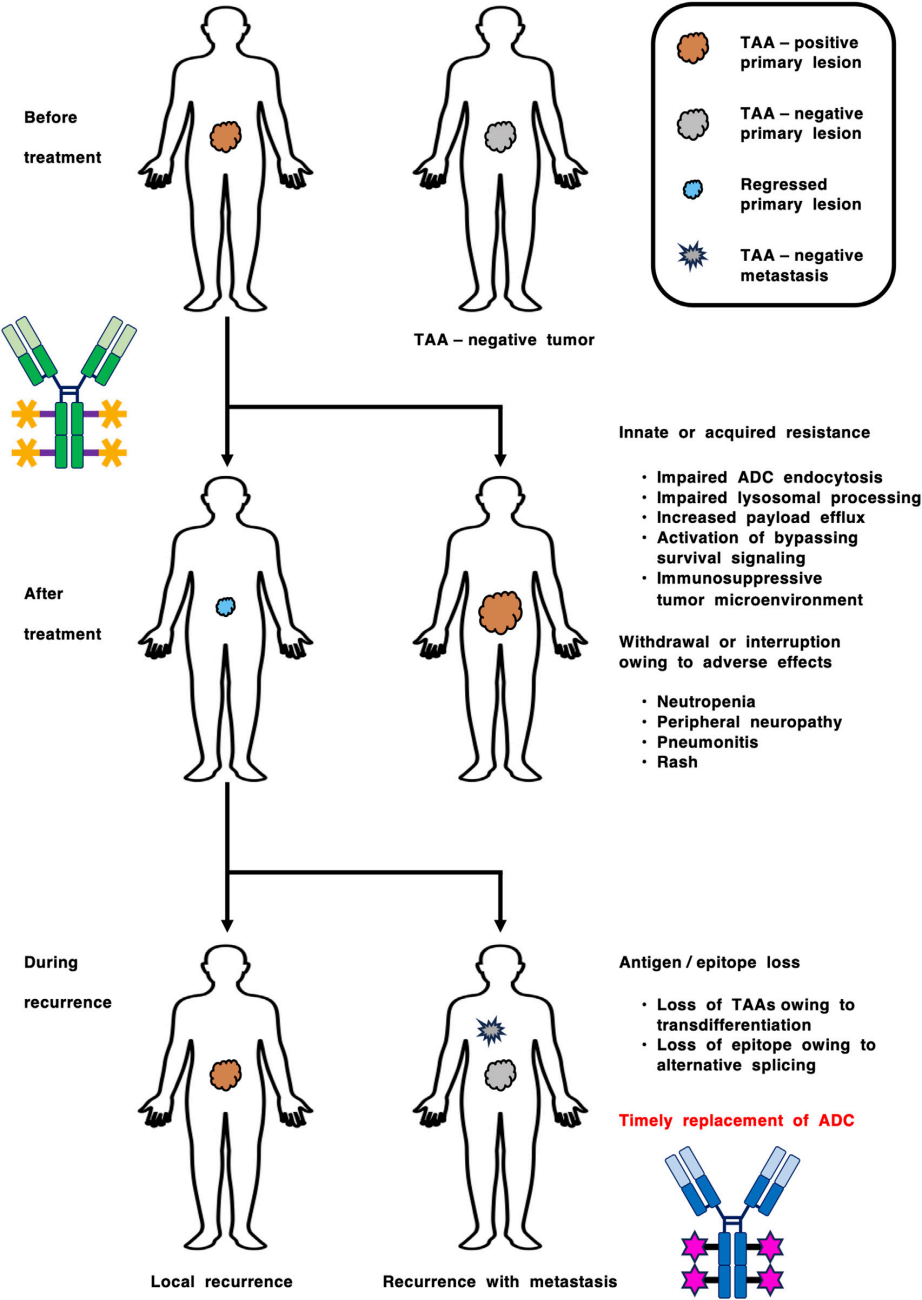
Longitudinal companion diagnostics for the optimization of antibody-drug conjugate (ADC) therapy. Innate and acquired resistance to ADCs are caused by various mechanisms, such as (1) inaccessibility of ADCs to tumor cells, (2) resistance to cytotoxic payloads, (3) loss of tumor-associated antigens (TAAs) owing to intra- or intertumor heterogeneity and transdifferentiation, and (4) loss of epitopes owing to alternative splicing. In contrast, adverse effects, such as neutropenia, peripheral neuropathy, pneumonitis (interstitial lung disease) and rash, lead to withdrawal or interruption of ADC therapy. Longitudinal monitoring of TAAs before treatment, after treatment and during recurrence could lead to improved clinical benefits via timely ADC replacement.

Innate and acquired resistance to ADCs are still poorly understood, but might be caused by various mechanisms, including (1) resistance to cytotoxic payloads, (2) inaccessibility of ADCs to tumor cells owing to barriers around or within the tumor microenvironment, and (3) the loss of antigens or epitopes on tumor lesions via intra- or intertumor heterogeneity, transdifferentiation and alternative splicing (Dumontet et al., 2023; Katoh et al., 2024b; Mosele et al., 2023; Nasiri et al., 2025). Immunohistochemical analyses in urothelial cancer patients receiving enfortumab vedotin treatment revealed that NECTIN4 antigen loss preferentially occurs in metastatic lesions compared with primary lesions (P < 0.001) and is associated with shortened PFS (P < 0.001) (Klümper et al., 2023). Owing to the NECTIN4 upregulation in luminal subtypes and downregulation in the neuroendocrine-like subtype as mentioned above (Chu et al., 2021), NECTIN4 antigen loss in the neuroendocrine-like subtype can induce innate resistance to enfortumab vedotin, whereas NECTIN4 antigen loss owing to transdifferentiation from the luminal to the endocrine-like subtype can lead to acquired resistance to enfortumab vedotin. In gastric cancer patients, CLDN18.2-staining intensity is decreased in peritoneal metastasis compared with primary lesion (Saito et al., 2025). Although further validation is necessary for generalization, these examples suggest that loss of TAAs, such as NECTIN4 and CLDN18.2, might occur in metastatic lesions owing to adaptation or plasticity of tumor cells in their metastatic niche. Longitudinal monitoring of TAAs before treatment, after treatment and during recurrence could lead to the optimal management of cancer via timely replacement of ADC (Figure 4).

## 6 Conclusion

ADCs, which target cell-cell adhesion molecules, constitute a rapidly emerging class of cancer therapeutics. Enfortumab vedotin is an FDA-approved NECTIN4-targeting ADC for urothelial cancer, and other NECTIN4-targeting drugs are in phase III clinical trials. Investigational anti-CNDN18.2 ADCs for the treatment of G/GEJAC patients and anti-CLDN6 and anti-CDH6 ADCs for the treatment of ovarian cancer have also proceeded to later-stage clinical trials. NECTIN4 and CLDN18.2 are “passenger” TAAs that are expressed in primary tumors but might be dispensable in metastatic niches, whereas RTKs, such as HER2 and MET, are oncogenic TAAs that drive tumorigenesis by themselves. TAA expression in primary *versus* metastatic tumors and introduction of bispecific, combination or sequential strategies are hot issues in this field. Bispecific ADCs and companion diagnostics are emerging to further improve the clinical benefits of adhesion molecule-targeted ADCs.
